# Modelling the longitudinal associations between schizotypy and aberrant salience: The role of mentalization and attachment

**DOI:** 10.1111/papt.12589

**Published:** 2025-03-21

**Authors:** Ercan Ozdemir, Angus MacBeth, Helen Griffiths

**Affiliations:** ^1^ Section of Clinical Psychology, School of Health in Social Science University of Edinburgh Edinburgh UK

**Keywords:** affective symptoms, attachment theory, mentalization, psychosis risk, schizotypy

## Abstract

**Objectives:**

This study investigates the role of mentalization and attachment in the development of schizotypy into aberrant salience. Specifically, we examine how disruptions in these socio‐cognitive capacities interact with multidimensional schizotypy to influence self‐fragmentation, emotional instability and social detachment.

**Method:**

Study variables were measured using self‐report scales. A two‐wave cohort study design was implemented with a 9‐month assessment interval. The cross‐sectional and longitudinal associations between mentalizing, attachment, negative affect, aberrant salience and schizotypy were estimated using network modelling.

**Results:**

The study involved 312 participants aged 18–37 years, with 77% identifying as female and 57% receiving mental health treatment during the follow‐up period. Results indicated that mentalizing capacity was central in linking concurrent negative affect, attachment and psychosis risk and served as a temporal bridge connecting multiple dimensions of psychosis risk.

**Conclusion:**

Mentalizing difficulties can heighten psychosis risk by maintaining affective dysregulation and consolidating schizotypy. The results may be influenced by potential sampling errors, as indicated by the analyses of network stability and accuracy. Interventions promoting mentalizing capacity may attenuate psychosis risk.

## INTRODUCTION

Psychosis risk manifests across different levels, from enduring personality organization, conceptualized as schizotypy (Fonseca‐Pedrero et al., 2021; Meehl, 1990), to states of aberrant salience (Kapur, 2003). Schizotypy encompasses three core dimensions: disorganized (e.g. incoherence of self‐experience), negative (e.g. social and emotional detachment) and positive (e.g. epistemic and perceptual aberrations). This triadic structure remains consistent across schizotypy, high‐risk states and active psychosis (Fonseca Pedrero & Debbané, 2017; Nelson et al., 2013; Rosell et al., 2014). Aberrant salience, characterized by heightened and dysregulated motivational salience, reflects destabilizing shifts in self‐ and world experiences that may precede psychosis onset (Kapur, 2003). However, the mechanisms driving the transition from schizotypy to aberrant salience remain partially understood. Emerging evidence suggests that disruptions in social cognitive capacities, particularly mentalization and attachment, may shape this progression by deepening self‐fragmentation, emotional instability and social detachment (Ozdemir et al., 2024). This study aims to examine how mentalizing and attachment capacities interact with multidimensional schizotypy to heighten psychosis risk.

Theoretical models of schizotypy position cognitive disorganization as its defining dimension, while negative schizotypy moderates the severity of clinical decompensation (Meehl, 1962, 1990). Supporting this framework, network analytic models identify schizotypal disorganization as the central dimension of schizotypy (Dodell‐Feder et al., 2019; Hasson‐Ohayon et al., 2018), a putative mediator of the relationship between negative and positive schizotypy dimensions (Christensen et al., 2019). While positive schizotypy predicts psychotic disorders in the general population, its predictive value diminishes in clinical high‐risk samples, where negative schizotypy more reliably identifies individuals at risk for transition to psychosis (Debbané et al., 2015). Furthermore, schizotypy dimensions are associated with different affective dynamics, whereby elevated schizotypal disorganization is associated with intensified negative affect (Hernández et al., 2023; Kemp et al., 2023) and elevated negative schizotypy is associated with diminished positive affect (Barrantes‐Vidal et al., 2015; Kwapil et al., 2012). Taken together, schizotypal disorganization may serve as a pivotal mechanism in the progression from subclinical vulnerability to full clinical decompensation, maintaining affective dysregulation and psychosis risk. Negative schizotypy, in turn, is associated with a poorer long‐term prognosis, whereas the predictive utility of positive schizotypy may depend on methodological and sample‐related factors.

Schizotypy encompasses a set of traits indicating proneness to psychosis, while aberrant salience is conceptualized as a pre‐psychotic state marked by anxiety‐inducing disruptions in meaning‐making and perceptual processes, which may culminate in psychosis (Kapur, 2003). Strong associations have been found between positive schizotypy and aberrant salience (Cicero et al., 2021; Poletti et al., 2022; Pugliese et al., 2022). Additionally, schizotypal disorganization not only bridges negative and positive schizotypy (Christensen et al., 2019) but also mediates the indirect effects of negative schizotypy on aberrant salience (Ozdemir et al., 2024). Moreover, the onset and persistence of aberrantly salient experiences may be influenced by premorbid cognitive and social functioning (Kapur, 2003), indicating that identifying relevant socio‐cognitive factors and promoting their functioning could potentially mitigate the trajectory toward psychosis.

Mentalization and attachment are socio‐cognitive processes that facilitate affect regulation and the construction of coherent self‐narratives (Fonagy et al., 2002). Mentalizing is a meaning‐making ability that enables individuals to understand and make sense of their own and others' thoughts, emotions and intentions. Disruptions in mentalization capacity often manifest as a stress‐reactive shift from reflective to automatic processing of mental states, resulting in biased interpretations that serve immediate emotional needs rather than fostering accurate self‐awareness (Luyten et al., 2020). Evidence indicates that mentalizing capacity is vulnerable to inhibition in the presence of negative affect (Taylor & Bagby, 2013), relational distress (Nolte et al., 2013) and psychotic disorganization (Bora et al., 2009; Sprong et al., 2007). Therefore, mentalizing impairment can be characterized by stress‐reactive diminishments in its capacity and the time to recovery from stress (Luyten et al., 2020).

Mentalizing impairments are closely linked to self‐disorders and social detachment that characterize the phenomenology of psychosis (Lysaker et al., 2020). These impairments are consistently associated with disorganization across the psychosis continuum (Lehmann & Ettinger, 2023; Myers et al., 2024), and robust associations are observed between mentalizing impairments and the presence of negative symptoms (McGuire et al., 2023). The negative dimension, in particular, appears to be related to an inability to mentalize affects, encompassing challenges in understanding both one's own emotions (Larøi et al., 2008; Martin et al., 2016; Prince & Berenbaum, 1993) and those of others (Kong et al., 2021; Sandor, 2022; Wang et al., 2020). Moreover, as disorganization intensifies, mentalizing capacity may further deteriorate, ultimately leading to a breakdown in reflective processing, with psychosis potentially emerging as a culmination of a collapse in mentalization (Liotti & Gumley, 2008).

The interplay between schizotypy and socio‐cognitive capacities can be further viewed through the lens of attachment theory, which conceptualizes attachment as an innate motivational system driving individuals to seek proximity to significant others for a sense of security in times of distress (Bowlby, 1980). The attachment system functions in tandem with mentalizing processes, such that secure attachment supports the capacity to reflect on mental states, fostering adaptive affect regulation and social understanding (Luyten et al., 2020). However, for individuals with insecure attachment, the activation of the attachment system, particularly in times of stress or relational distress, can inhibit mentalizing, leading to biased interpretations of mental states. Disruptions in the attachment system manifest in three profiles: anxiety, avoidance and disorganization (Mikulincer & Shaver, 2016). Attachment anxiety is marked by heightened stress reactivity and an intense need for proximity to others as a means of distress regulation, while an avoidant profile is characterized by hypo‐responsivity to stress and over‐reliance on autonomous affect regulation (Luyten et al., 2020). Attachment disorganization, on the contrary, is indicated by the simultaneous activation of anxiety and avoidance underlying the absence of an organized affect regulation strategy.

Systematic reviews highlight significant associations between attachment avoidance and negative symptoms and between attachment anxiety and positive symptoms in psychosis (Gumley et al., 2014; Herstell et al., 2021; Korver‐Nieberg et al., 2014; van Bussel et al., 2021). Although associations between attachment disorganization and psychotic phenomenology were not reviewed in these studies, other research reveals that attachment disorganization is linked to both negative and positive schizotypy (Sheinbaum et al., 2013). Therefore, attachment avoidance may contribute to social and emotional detachment associated with negative schizotypy, and attachment anxiety may be implicated in psychotic stress reactivity, while attachment disorganization may influence both social aversion and stress reactivity.

Accordingly, disruptions in mentalization and attachment seem to play a critical role in the development of psychosis by reinforcing schizotypy and linking its dimensions to affective dysregulation and aberrant salience. Despite this, a comprehensive assessment of the interactions between these constructs remains limited. To address this gap, the current study aimed to analyse the concurrent and temporal relationships among schizotypy, aberrant salience, negative affect, attachment and mentalization. We hypothesized that mentalizing and attachment capacities would serve as mediators, connecting schizotypy to aberrant salience.

## METHODS

### Design

A two‐wave longitudinal cohort study was conducted with ethical approval obtained from the University of Edinburgh Health in Social Science ethics committee (ref: CAHSS 2205/05). The inclusion criteria were being aged between 18 and 35 years, and English fluency. The exclusion criteria were experiencing a mental health crisis or severely low mood. Online advertisements were utilized to recruit participants from the general population. Recruitment was restricted to individuals residing in English‐speaking countries, including Australia, Canada, Ireland, New Zealand, South Africa, the United Kingdom and the United States. The baseline sample included 1263 volunteers who completed data collection from September to December 2021. Further information about sampling and characteristics of the participant pool is reported in Ozdemir et al. (2024). Follow‐up recruitment occurred from July to September 2022, with 1105 volunteers who had given informed consent invited to participate. On average, participants completed the follow‐up survey approximately nine months after baseline (M_days_ = 286.58, SD_days_ = 27.47), a time frame commonly employed in previous research to examine short‐term longitudinal associations between psychosis and socio‐cognitive functioning (Cowman et al., 2021).

### Measures

Demographic questions measured age, gender and mental health. Mental health questions asked about the participant's lifetime history of psychosis (i.e. Have you ever experienced a psychotic episode?), current treatment for mental health issues (i.e. Are you currently receiving mental health treatment?) and any history of psychosis in their immediate family (i.e. Does anyone from your immediate family have a diagnosis of a psychotic disorder?). The mental health questions were responded to categorically with the options ‘Yes’, ‘No’, ‘Prefer not to say’.

#### Schizotypy

Schizotypy was assessed using the Multidimensional Schizotypy Scale‐Brief (MSS; Gross et al., 2018). The MSS is a 38‐item measure rated on a dichotomous scale and operationalizes schizotypy along the negative, disorganized and positive dimensions. Negative schizotypy was defined by social anhedonia and diminished emotional experiences. Disorganized schizotypy assessed incoherence in cognition and behaviour, and positive schizotypy involved unusual experiences such as magical ideation and perceptual aberrations. The convergent validity of the MSS is supported by its associations with interview‐rated assessments of schizotypy (Kemp et al., 2020). Schizotypy was included in the analyses as a multidimensional construct, and its negative (*α*_t1_ = .85, *α*_t2_ = .82), disorganized (*α*_t1_ = .90, *α*_t2_ = .92) and positive (*α*_t1_ = .78, *α*_t2_ = .83) dimensions showed acceptable reliability.

#### Aberrant salience

Aberrant salience was measured using the Aberrant Salience Inventory (ASI; Cicero et al., 2010). The ASI measures psychosis risk states based on alterations in self‐experiences in five domains via 29 items rated on a dichotomous scale. These domains involve (1) increased significance of usual stimuli, (2) perceptual changes marked by a feeling of heightened acuity of the senses, (3) cognitive changes pertaining to meaning‐making processes, (4) philosophical re‐orientation and (5) heightened emotionality. The ASI was reported to show acceptable convergent validity, indicated by associations with various dimensions of psychosis risk. Aberrant salience was included in the models as a unidimensional construct and was reliable in the baseline (*α* = .92) and follow‐up assessments (*α* = .93).

#### Mentalizing

The uncertainty about mental states scale of the Reflective Functioning Questionnaire‐8 (RFQ; Fonagy et al., 2016) was used to examine mentalizing difficulties. The RFQ is an eight‐item measure rated on a seven‐point scale ranging from 1 (strongly disagree) to 7 (strongly agree). The RFQ's mental state uncertainty scale examines difficulties in attributing mental states to others, understanding motivations guiding one's behaviour and emotional reactivity. The uncertainty scale displays robust associations with self‐harm, psychosis‐like experiences and affective dysregulation, supporting its construct validity (Fonagy et al., 2016). The reliability of the mental state uncertainty scale was questionable at baseline (*α* = .69) and acceptable at follow‐up (*α* = .83).

#### Attachment

Anxiety, avoidance and disorganization dimensions of attachment were assessed using the Revised Psychosis Attachment Measure (PAM; Pollard et al., 2020). The PAM is a 26‐item instrument scored on a 4‐point scale ranging from 0 (not at all) to 3 (very much). Attachment anxiety is characterized by an excessive dependence on others to regulate distress and fears of abandonment and rejection, whereas attachment avoidance reflects an exaggerated self‐reliance on regulating distress. The attachment disorganization subscale assesses fears of intimacy, relational ambivalence and the lack of a coherent strategy to regulate distress. Psychometric evaluations of the PAM have shown mixed results regarding its dimensional structure. While a three‐dimensional model was supported by a psychometric study (Justo‐Nunez et al., 2024), other studies have reported issues, particularly with the attachment avoidance dimension (Bussel et al., 2024; Olbert et al., 2016). Despite these inconsistencies, the three‐dimensional PAM was utilized in this study due to its relevance in capturing attachment‐related processes in the context of psychosis phenomenology. Disorganization (*α*_t1_ = .90, *α*_t2_ = .92), avoidance (*α*_t1_ = .86, *α*_t2_ = .87) and anxiety dimensions (*α*_t1_ = .85, *α*_t2_ = .86) of the PAM showed acceptable reliability.

#### Negative affect

The Depression Anxiety Stress Scale‐21 (DASS; Antony et al., 1998) was used to assess negative affective states over a one‐week period. The DASS is a four‐point rating scale of depression, anxiety and stress, each assessed with seven items. The three‐dimensional structure, validity and reliability of the DASS were supported by psychometric studies (Brown et al., 1997; Zanon et al., 2021). The depression (*α*_t1_ = .91, *α*_t2_ = .91), anxiety (*α*_t1_ = .83, *α*_t2_ = .86) and stress (*α*_t1_ = .84, *α*_t2_ = .88) dimensions of the DASS were reliable and included in the models.

### Data analysis

Analyses were conducted in RStudio 2023.06.1 (Posit team, 2023). The indices of schizotypy, aberrant salience, mentalization, attachment and negative affect were compared between baseline and follow‐up assessments using paired‐samples *t*‐tests. Additionally, independent‐samples Mann–Whitney *U* tests were conducted to compare participants with and without a history of psychosis, and Kruskal–Wallis tests were conducted to investigate gender differences in baseline variables. Network modelling was utilized to investigate both cross‐sectional and longitudinal relationships between the variables collected at baseline and follow‐up. The recommended sample size to observe moderate sensitivity and high specificity and edge weight correlations for sparse cross‐sectional networks with fewer than 20 nodes ranges from 250 to 350 (Constantin & Cramer, 2022).

#### Contemporaneous networks

Cross‐sectional partial correlations between the study variables were estimated via contemporaneous network modelling using the ‘bootnet’ package (Epskamp & Fried, 2018). The networks were visualized using the ‘qgraph’ package (Epskamp et al., 2012). Given the non‐normally distributed ordinal data, Spearman's correlation coefficients were used to estimate the networks. The graphical least absolute shrinkage and selection operator estimator with Extended Bayesian Information Criterion (EBICglasso; Tibshirani, 1996) was applied for regularization, which refines the model to produce a sparse network by pruning weaker edges.

The ‘qgraph’ package was used to plot the strength centrality indices, which reflect the overall magnitude of associations between a specific variable and all other variables in the model (Robinaugh et al., 2016). Bridge centrality indices were also estimated via the ‘networktools’ package (Jones, 2022) to measure how strongly a variable connects other variables in the network. To compare the baseline and follow‐up networks, a permutation‐based approach was implemented using the ‘NetworkComparisonTest’ package (Van Borkulo et al., 2023). This comparison assessed differences in global strength, edge weights and centrality estimates across the two time points. The ‘bootnet’ package (Epskamp et al., 2018) was used to evaluate edge‐weight accuracy and stability of centrality estimates. The accuracy of the edge weights was estimated via a case‐drop bootstrap method. A subset bootstrap method was applied to assess the stability of centrality estimates via the metric of correlation stability coefficient (CSC), with values below 0.25 indicating unacceptable stability and above 0.5 indicating acceptable stability (Epskamp et al., 2018).

#### Temporal networks

A cross‐lagged panel network model (CLPN; Wysocki et al., 2022) was fitted to estimate the longitudinal associations between the study variables. The CLPN model estimates directed associations between variables across time points, specifically between a variable at time t and the same or other variables at time *t* + 1, while controlling for all other variables at time *t*. A hybrid estimation approach was applied, in which a preliminary model was estimated via lasso regression – a regularization technique that shrinks smaller coefficients to zero – then re‐estimated via structural equation modelling. This two‐step estimation approach results in a sparse and parsimonious network.

The centrality of the variables in the network was evaluated based on cross‐lagged in‐ and out‐prediction estimates. The cross‐lagged in‐prediction is a metric that indicates the extent to which a follow‐up variable is influenced by all baseline variables, excluding the variable's own autoregressive effect. By contrast, cross‐lagged out‐prediction refers to the average influence of a baseline variable on all other follow‐up variables, calculated by averaging the directional effects while excluding its autoregressive effect. The stability of these centrality metrics was assessed using a non‐parametric bootstrapping approach, drawing 1000 samples the same size as the original sample. The bootstrapping procedure sets confidence intervals around the in‐ and out‐prediction estimates, with smaller confidence intervals indicating the stability of the estimates.

## RESULTS

### Sample characteristics

Study invitations were sent to 1105 of the baseline volunteers, 551 of whom consented to participate in the follow‐up assessment. Participants were excluded due to duplicate responses (*n* = 21), consenting but not participating (*n* = 47), data linkage issues (*n* = 102) and partial completion (*n* = 69). Missing data occurred at the scale level rather than individual items, with the highest attrition observed for the RFQ, the final scale in the measurement battery, likely due to participant burden. The final sample included 312 participants who provided complete research data. At follow‐up, participants were aged between 18 and 37 years (*M* = 28.93, SD = 4.41); 77% (*n* = 240) were female, 89% (*n* = 278) were White and 60% (*n* = 187) were employed. History of psychosis was self‐reported by 30% (*n* = 94), and family history of psychosis was reported by 16% (*n* = 50) of the sample. Fifty‐seven percent of the sample (*n* = 178) reported undergoing mental health treatment. Compared with the cut‐off value, the ASI indices observed in the sample indicated the prevalence of aberrantly salient experiences both in the baseline (*M* = 13.5, SD = 7.39) and follow‐up (*M* = 13.6, SD = 7.74) assessments based on meta‐analytical evidence for an index of 13.5 as the optimal cut‐off threshold for psychosis proneness (Merola et al., 2023).

Comparisons of the variables across both assessment points indicated elevated indices of schizotypal disorganization, aberrant salience, stress and mental state uncertainty in follow‐up compared with the baseline assessment (see Table 1). Compared to participants without a history of psychosis, participants with previous experience of psychosis scored significantly higher on all study variables at baseline assessment, other than avoidant attachment and negative schizotypy (see Table 2). Furthermore, statistically significant gender differences in the baseline variables were found (see Table 3). Positive and negative schizotypy and aberrant salience levels were higher in male participants compared with female participants. Conversely, attachment anxiety and disorganized schizotypy levels were higher in female participants compared with male participants.

**TABLE 1:** Paired‐samples *t*‐test results for the differences between baseline and follow‐up values of the study variables.

	*M*_t1_ (SD_t1_)	*M*_t2_ (SD_t2_)	*t*	*p*	Cohen's *d*
Negative schizotypy	3.56 (3.39)	3.47 (3.15)	0.68	.494	.04
Disorganized schizotypy	4.45 (3.94)	5.22 (4.32)	−4.57	<.001	−.25
Positive schizotypy	3.26 (2.95)	3.49 (3.21)	−1.89	.059	−.11
Aberrant salience	13.62 (7.42)	15.12 (3.73)	−5.06	<.001	−.30
Stress	9.86 (4.77)	10.44 (5.31)	−2.18	<.05	−.12
Anxiety	6.96 (4.81)	7.04 (5.05)	−0.32	.750	−.02
Depression	9.15 (5.73)	9.36 (5.84)	−0.65	.513	−.03
Attachment disorganization	14.92 (8.04)	14.82 (8.54)	0.29	.776	.02
Attachment avoidance	12.51 (4.95)	12.42 (5.17)	0.41	.680	.02
Attachment anxiety	14.69 (5.75)	14.74 (5.98)	−0.16	.873	.00
Mental state uncertainty	1.06 (0.64)	1.18 (0.80)	−3.38	<.001	−.56

**TABLE 2:** Mann–Whitney *U* Test results for the differences in the baseline study variables between participants with and without a history of psychosis.

	Psychosis history		*U*	*p*
	Yes (*n* = 92) mean rank	No (*n* = 208) mean rank		
Negative schizotypy	161.34	149.29	9133.5	.266
Disorganized schizotypy	194.73	134.41	5994	<.001
Positive schizotypy	204.84	129.91	5044.5	<.001
Aberrant salience	199.69	132.20	5528.5	<.001
Stress	180.02	140.96	7377.5	<.001
Anxiety	183.52	139.41	7048.5	<.001
Depression	171.22	144.66	8157.5	<.001
Attachment disorganization	179.16	141.35	7458	<.001
Attachment avoidance	160.21	149.79	9239.5	.340
Attachment anxiety	189.94	136.55	6445	<.001
Mental state uncertainty	195.69	133.98	5904.5	<.001

**TABLE 3:** Kruskal–Wallis test results of significant gender differences in baseline variables.

Variable	Gender categories		Rank difference	SE	*p* *
Stress	Male	Female			
		Non‐binary			
	Female	Non‐binary	−41.51	16.43	<.05
Aberrant salience	Male	Female	39.61	16.25	<.05
		Non‐binary			
	Female	Non‐binary	−45.37	16.45	<.05
Attachment anxiety	Male	Female	−31.83	16.24	<.01
		Non‐binary			
	Female	Non‐binary			
Negative schizotypy	Male	Female	47.38	16.11	<.05
		Non‐binary			
	Female	Non‐binary			
Disorganized schizotypy	Male	Female			
		Non‐binary	−53.74	21.45	<.05
	Female	Non‐binary	−89.26	16.34	<.001
Positive schizotypy	Male	Female	46.65	17.39	<.05
		Non‐binary			
	Female	Non‐binary			

* Adjusted significance level based on Bonferroni correction.

### Contemporaneous networks

**FIGURE 1:**
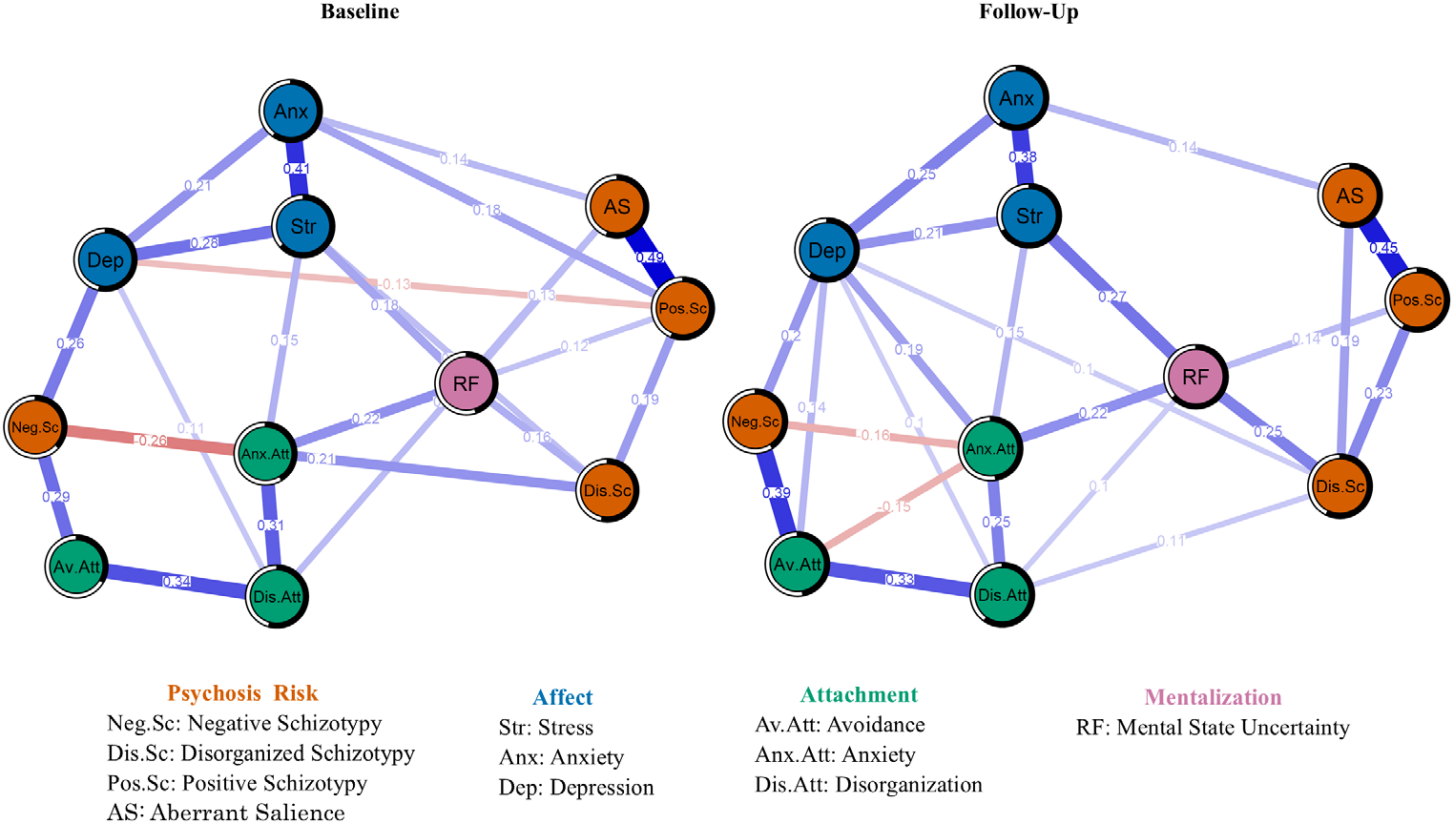
Cross‐sectional networks of psychosis risk, mentalizing, attachment and negative affect at baseline and follow‐up assessments.

**FIGURE 2:**
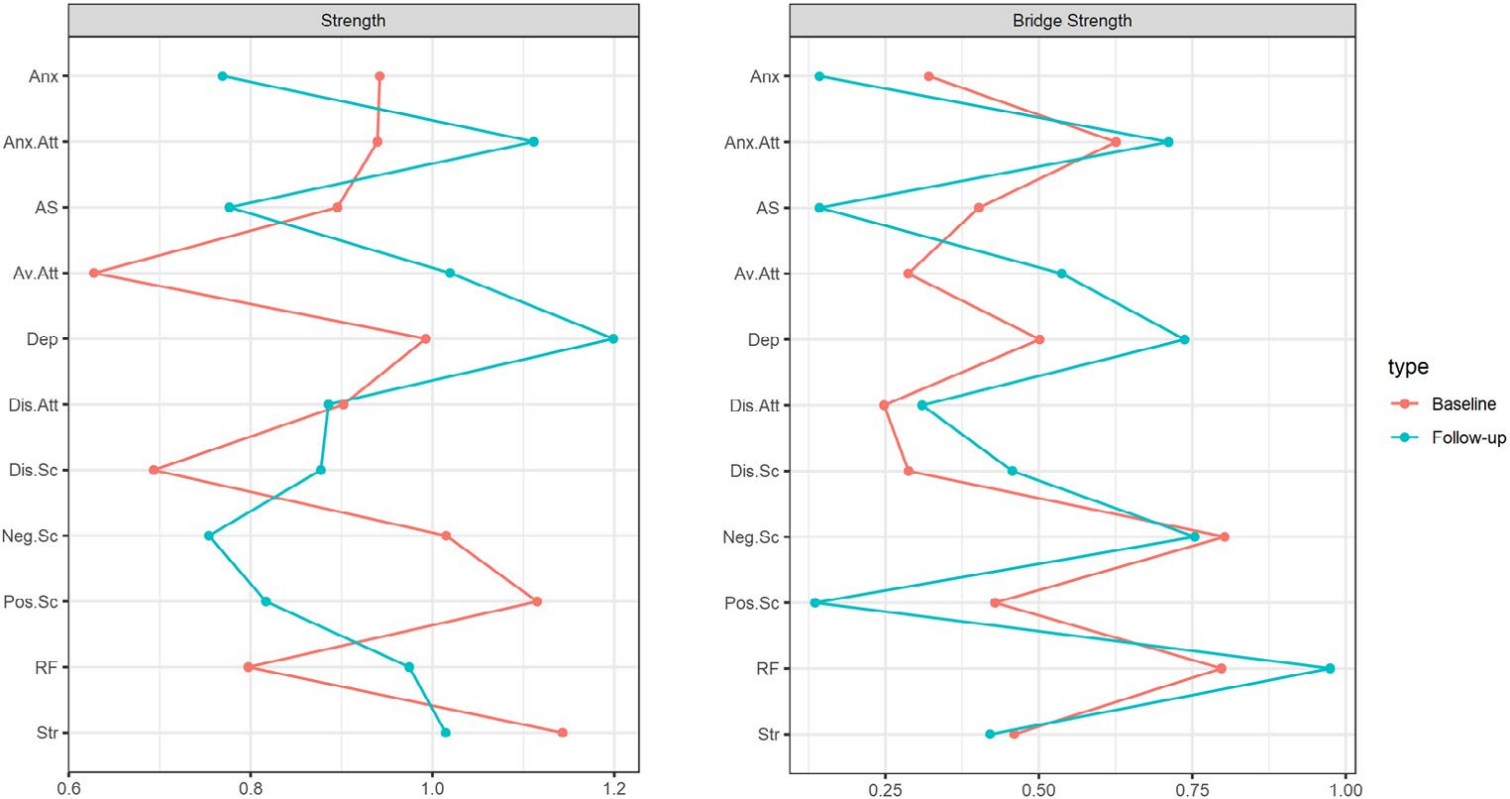
Strength and bridge strength centrality indices for the baseline and follow‐up networks.

Figure 1 illustrates the contemporaneous networks of psychosis risk and affective dysregulation. Of the 55 potential associations, 23 were statistically significant in the baseline network and 24 associations in the follow‐up network. Among all variables, stress consistently exhibited the highest predictability (see Table 4). Centrality indices are detailed in Figure 2, with stress and positive schizotypy demonstrating the highest strength centrality in the baseline network. By contrast, depression and attachment anxiety showed the highest strength centrality in the follow‐up network. The key bridging variables across assessments were mental state uncertainty (bridge_baseline_ = 0.79, bridge_follow‐up_ = 0.97) and negative schizotypy (bridge_baseline_ = 0.80, bridge_follow‐up_ = 0.75). Mental state uncertainty served as a bridge linking psychosis risk dimensions with stress and attachment anxiety, while negative schizotypy connected depression and avoidant attachment.

**TABLE 4:** Predictability estimates of the study variables.

Variables	*R* ^2^_baseline_	*R* ^2^_follow‐up_
Negative schizotypy	.37	.41
Disorganized schizotypy	.53	.57
Positive schizotypy	.56	.58
Aberrant salience	.56	.55
Stress	.63	.67
Anxiety	.60	.59
Depression	.54	.59
Attachment disorganization	.54	.60
Attachment avoidance	.33	.47
Attachment anxiety	.41	.47
Mental state uncertainty	.45	.63

Reliability of the results was evaluated based on network structure invariance across time points, and stability and accuracy of the estimates. Overall strength of the associations within the networks remained consistent between the baseline and follow‐up assessments as indicated by a network comparison test, suggesting structural invariance (*S* = 0.07, *p* = .88). Notably, attachment avoidance was the sole strength centrality index that differed significantly between the two time points. Additionally, the association between negative and disorganized schizotypy was significant only at baseline.

Bootstrap analyses indicated questionable stability and accuracy of the network's edge estimates (Figure S1 provides a visual representation of the accuracy analyses). Using a non‐parametric bootstrapping procedure with 5000 samples, the results suggested reliable edge weights. However, the stability analysis indicated questionable reliability in centrality estimates for the baseline network (CS‐bridge strength = 0.21, 95% CI = [0.13, 0.28]; strength = 0, 95% CI = [0, 0.13]). For the follow‐up network, the bridge centrality estimates were relatively stable (CS‐bridge = 0.44, 95% CI = [0.36, 0.52]), while the strength centrality estimates were unreliable (strength = 0, 95% CI = [0, 0.05]). Overall, the reliability analysis indicates a potential sampling error.

### Temporal network

**FIGURE 3:**
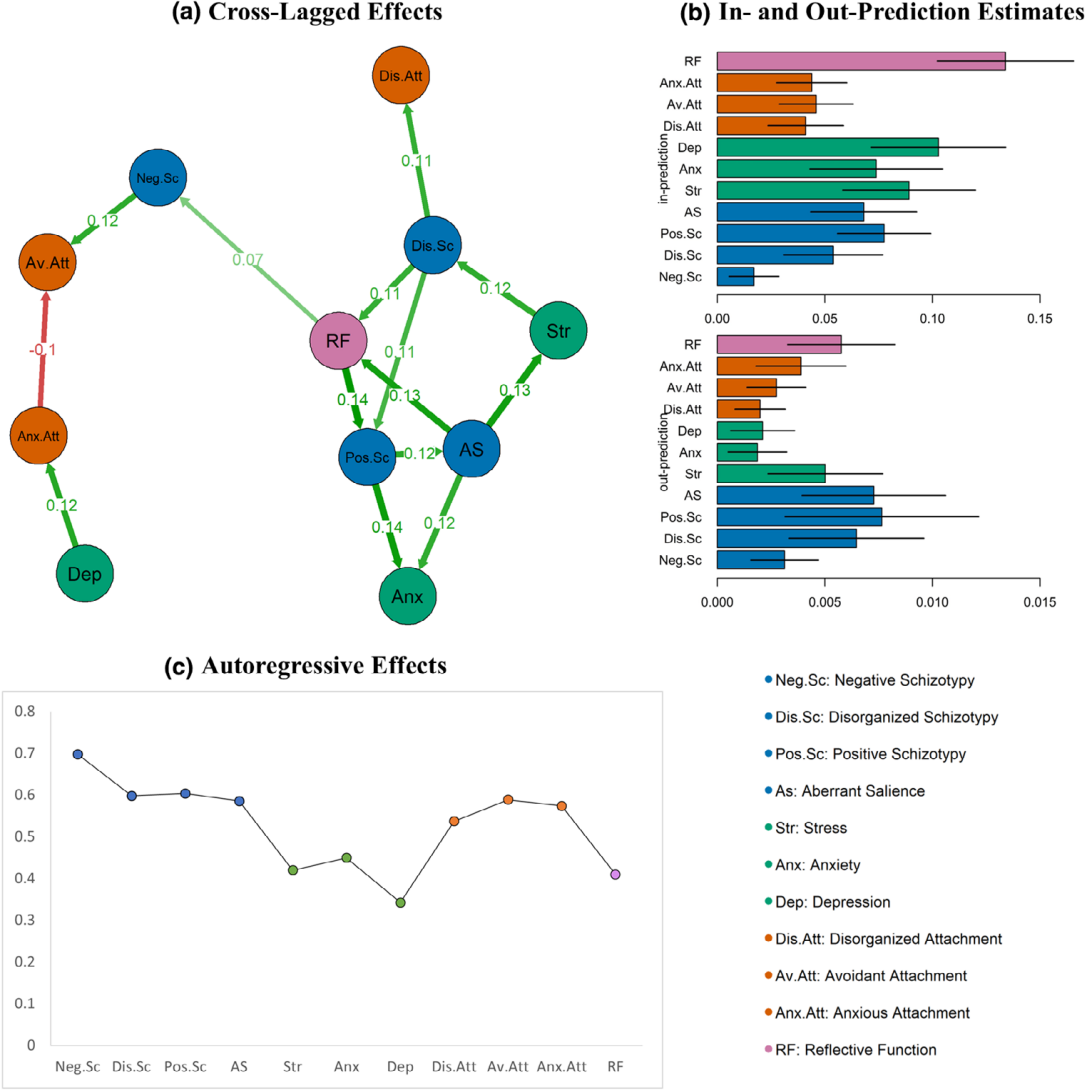
The temporal network. (a) Demonstrates the temporal network model of negative affect, attachment, mentalization and psychosis risk. (b) contains the bootstrapped influence (out‐prediction) and predictability (in‐prediction) indices. (c) is the line chart of autoregressive effect estimates for each node grouped according to each construct.

The CLPN model is illustrated in Figure 3a. Results indicated that mental state uncertainty is a bridge between psychosis risk dimensions, transmitting the indirect effects of disorganized schizotypy on aberrant salience and negative and positive schizotypy. Of note, elevated schizotypy indices at baseline predicted heightened negative affect and attachment insecurity, and disorganization 9 months later. Bootstrapped cross‐lagged in‐prediction estimates identified mental state uncertainty and depression as the most strongly predicted nodes in the network. Conversely, bootstrapped out‐prediction indices revealed that aberrant salience, along with disorganized and positive schizotypy dimensions, were the most influential nodes. However, confidence intervals for the indices of these highly influential nodes suggested low stability in their predictive effect sizes (see Figure 3b). The autoregressive effects indicated that psychosis risk and attachment nodes were persistent over a 9‐month period, whereas mental state uncertainty and negative affect showed more variability (see Figure 3c).

## DISCUSSION

The current study aimed to examine the unfolding of schizotypal experiences into aberrant salience, hypothesizing that disruptions in mentalizing and attachment capacities could accelerate this progression by worsening self‐fragmentation, emotional instability and social detachment, thereby heightening psychosis risk. The findings underscore the central role of mentalizing capacity in the temporal progression of psychosis risk. Network models indicated that mentalizing difficulties not only link different dimensions of psychosis risk over time but also serve as a bridge between affective dysregulation and psychosis risk at each time point. By contrast, while attachment capacity played a more peripheral role, notable associations emerged: attachment anxiety was associated with concurrent negative affect and mentalizing difficulties, whereas attachment avoidance and disorganization were temporally influenced by negative and disorganized schizotypy, respectively. Given that attachment and mentalizing operate as loosely coupled systems (Fonagy & Bateman, 2016), these findings suggest that, within an attachment and mentalization‐informed model, mentalization disruptions, rather than attachment, may act as the primary mechanism underpinning psychosis risk.

Building on the role of mentalizing and attachment capacities in potentiating psychosis risk, the current study further investigates how disorganized and negative schizotypy consolidates psychosis risk. Research from high‐risk paradigms indicates that disorganized and negative experiences predict psychosis onset but remain under‐researched dimensions of psychosis risk (Fusar‐Poli et al., 2020; Oliver et al., 2020; Simon et al., 2013). Addressing this gap, the current study implicates disorganized and negative schizotypy in different dynamics interactively contributing to psychosis development.

Schizotypal disorganization is considered a fundamental dimension of the psychosis spectrum (e.g. Meehl, 1990) influencing affective dynamics (Hernández et al., 2023; Kemp et al., 2023) and mentalizing processes (Bora et al., 2009; Sprong et al., 2007). Estimating these associations via network modelling, the present study revealed that disorganized schizotypy predicts increased mentalizing difficulties, positive schizotypy and attachment disorganization over time. The results provide support for an attachment model postulating psychosis as the outcome of the collapse of self‐reflective capacity and attachment organization due to acute experiences of disorganization (Liotti & Gumley, 2008). Therefore, schizotypal disorganization may be a maintaining factor of psychotic reactivity to stress.

The present study further elucidates the role of negative schizotypy within the interconnected dynamics of affective and social processes, particularly in relation to depression and attachment avoidance. The estimated associations align with existing research that links negative schizotypy to diminished positive affect (Barrantes‐Vidal et al., 2015; Kwapil et al., 2012) and negative symptoms to avoidant attachment (Gumley et al., 2014; Korver‐Nieberg et al., 2014). Social anhedonia is a common dimension shared among negative schizotypy and depression (Krynicki et al., 2018), showing particular associations with poor quality of life (Li et al., 2023; Ritsner et al., 2018; Wong et al., 2024). Additionally, the present study suggested that negative schizotypy could predict attachment avoidance, which may in turn reinforce the barriers to help‐seeking (e.g. MacBeth et al., 2013; Skrobinska et al., 2024; Sood et al., 2022). Therefore, negative schizotypy may sustain deactivating affect regulation patterns involving avoidance of emotional experiences and perceptions of others as unreliable sources of support and could be a central quality implicated in therapeutic outcomes.

The therapeutic mechanisms of change have been largely unknown for both the negative (Galderisi et al., 2021; Riehle et al., 2020) and disorganized (Wiesepape et al., 2023) dimensions of psychosis. Mentalization has been proposed as a central construct in various theoretical models of psychosis (Liotti & Gumley, 2008; Lysaker et al., 2020; Weijers et al., 2020), and our results suggest that facilitating the functioning of mentalizing processes involved in affect regulation and self‐organization could reduce psychosis risk by fostering coherence to narrative self‐experiences and a sense of social relatedness.

Our results should be interpreted with caution due to several methodological limitations. Although the research sample was identified as psychometrically psychosis‐prone based on the ASI's cut‐off point (see; Merola et al., 2023), stability tests of the centrality estimates showed questionable values, suggesting a potential sampling error, possibly arising from the underrepresentation of male participants in the sample. This may limit the reliability and generalizability of the findings. Additionally, self‐report assessment of mentalization and attachment may not fully capture the implicit, stress‐reactive processes that influence these capacities. For example, self‐report attachment measures may reflect profiles of interpersonal emotion regulation rather than the underlying implicit processes of perceptual defense or vigilance (Ravitz et al., 2010). In terms of mentalization, the RFQ includes items that capture aspects of affective dysregulation, which may confound the measurement of mentalizing capacity and inflate the estimated effect sizes. Several methodological refinements could enhance the quality of the findings. These may involve examination of the variables over a longer period with more frequent assessment points, implementing mixed methods to record performance‐ and interview‐based indices of mentalization and attachment. The stress‐reactive nature of mentalization capacity could be further explored using momentary assessment methods. Furthermore, future research could benefit from integrating network theory (e.g. Borsboom, 2017) to better capture the complex, dynamic interplay between mentalization, attachment and schizotypy, moving beyond static models to reflect the evolving nature of these psychological processes over time.

## CONCLUSION

The findings of this study offer important theoretical and clinical contributions by mapping dynamic interactions between affective dysregulation and psychosis risk, with a particular focus on the mediating role of mentalization. The identification of mentalization as a core component that interlinks various dimensions of psychosis risk enhances our understanding of how self‐disorders and social detachment manifest and persist in psychosis‐prone individuals. Clinically, these insights emphasize the need for targeted interventions that specifically address mentalization impairments to mitigate psychosis risk. While attachment capacity played a limited role, the observed temporal effects associated with attachment avoidance and disorganisation suggest specific pathways through which schizotypal features influence social functioning over time. These findings indicate that facilitating a balanced mentalizing capacity could be a therapeutic agent improving outcomes for individuals at risk of psychosis.

## AUTHOR CONTRIBUTIONS

**Ercan Ozdemir:** Conceptualization; methodology; data curation; formal analysis; writing – original draft; writing – review and editing. **Angus MacBeth:** Conceptualization; methodology; supervision; writing – review and editing. **Helen Griffiths:** Conceptualization; methodology; supervision; writing – review and editing.

## CONFLITS OF INTEREST STATEMENT

All authors declare that they have no conflits of interest.

## Supporting information


Data S1.

## Data Availability

The data that support the findings of this study are available from the corresponding author upon reasonable request.
