# Supplementary material for: Modelling the longitudinal associations between schizotypy and aberrant salience: The role of mentalization and attachment
Source: Psychol Psychother. 2025 Mar 21;98(3):763–78. doi: 10.1111/papt.12589 (PMC12346266; doi:10.1111/papt.12589)
Supplement: Supplementary file 1 — Data S1. [file PAPT-98-763-s001.zip › papt12589-sup-0002-FigureS1.docx]

**SUPPLEMENTARY MATERIALS**

**Follow-up** Edge stability of the follow-up network


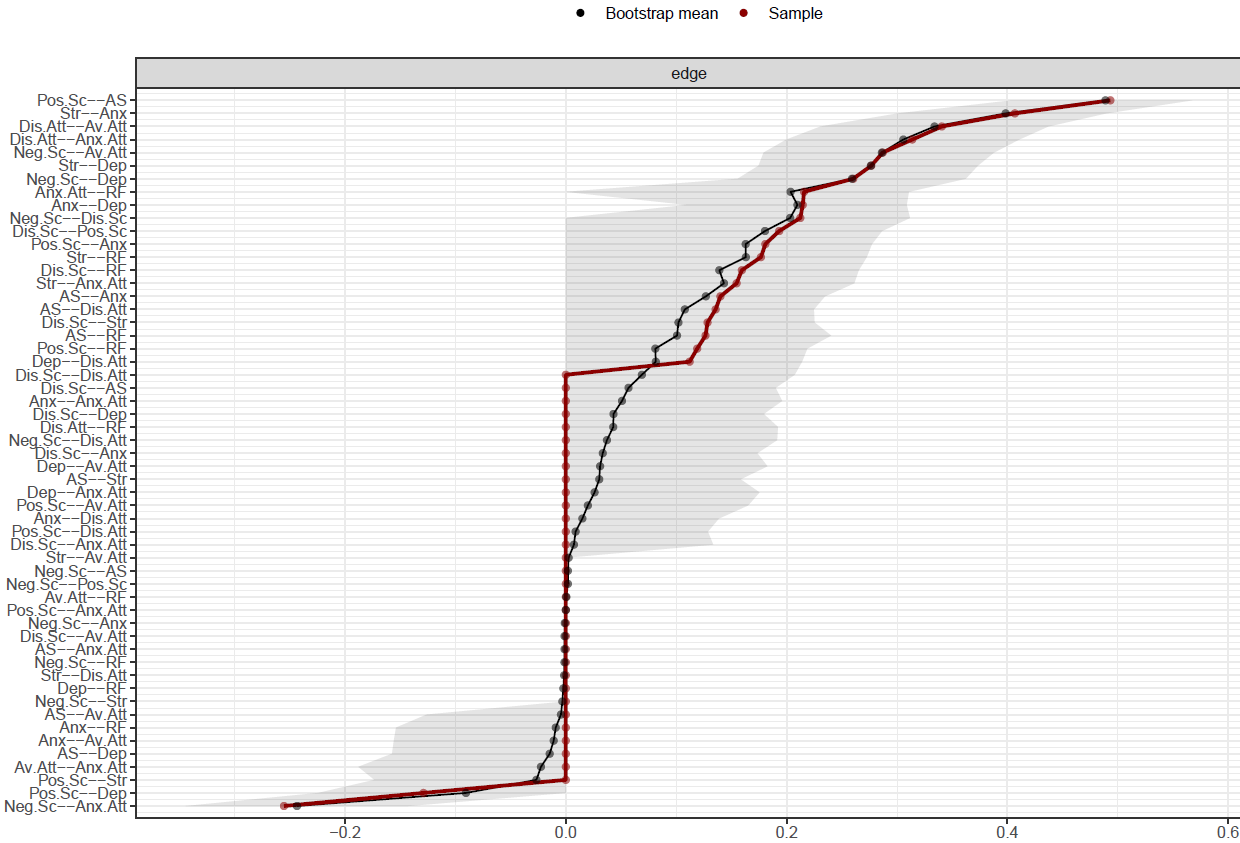


**Baseline**


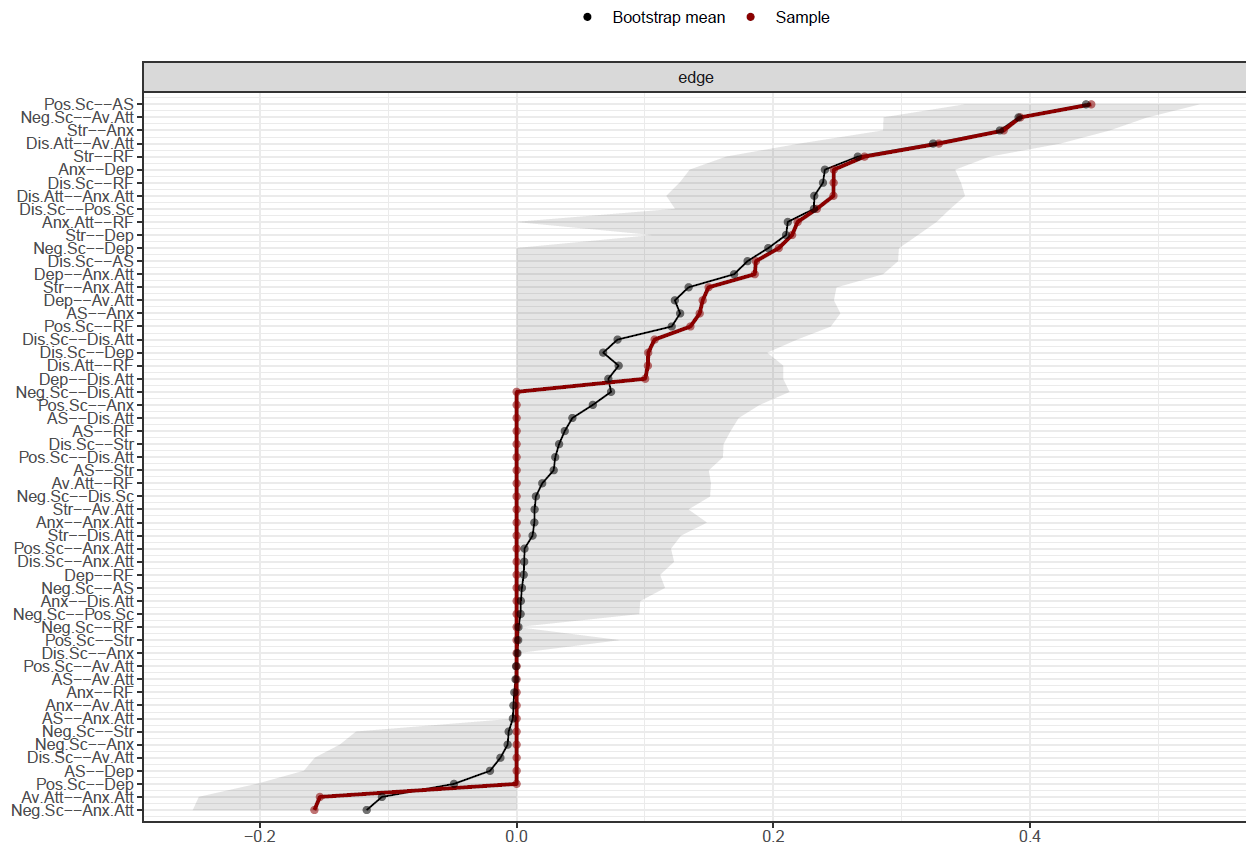


**Figure S1** Edge weigh accuracy based on case-drop bootstrapping
